# Activated autologous dendritic cells injected intratumorally are able to overcome local and systemic immune suppression imposed by the tumor and its microenvironment

**DOI:** 10.1186/2051-1426-2-S3-P61

**Published:** 2014-11-06

**Authors:** Vivek Subbiah, Omar Kayaleh, Ravi Murthy, David Hong, Siqing Fu, Aung Naing, Anthony Conley, Chitra Hosing, Indreshpal Kaur, Robert Prins, Funda Meric-Bernstam, Marnix Bosch

**Affiliations:** 1 Department of Investigational Cancer Therapeutics, University of Texas M.D. Anderson Cancer Center Houston, TX, USA; 2 Orlando Health, FL, USA; 3 University of Texas M.D. Anderson Cancer Center Houston, TX, USA; 4 ULCA, Los Angeles, CA, USA; 5 Northwest Biotherapeutics, USA

## Introduction

Cancer vaccines aiming to induce anti-tumor immune responses are generally considered most effective in an environment with minimal residual disease, due to the immunosuppressive effects exerted by most cancers. We hypothesized that appropriately activated autologous dendritic cells (DCVax^®^-Direct) may be able to overcome this suppressive environment through the production of inflammatory cytokines and through the activation of signal transduction pathways that condition the dendritic cells for completion of the maturation processes even after intratumoral injection.

## Methods

A Phase I trial that enrolled 36 patients with diverse solid tumor cancers. Three intratumoral DCVax-Direct injections were given at Day 0, 7 and 14, followed by additional injections at weeks 8, 16 and 32. Patients were followed for safety parameters, for tumor response using appropriate imaging, and for pathological evaluation of tumor tissue from serial biopsies. Evaluation of circulating immune cell populations as well as circulating cytokines is in progress.

## Results

Recurrent low to moderate fevers were seen in most patients following DCVax-Direct administration. Substantial apparent increases in tumor size, were observed on CT or PET scans in the majority of patients. Tumor biopsies of injected lesions demonstrated extensive tumor necrosis, as well as large immune cell accumulations, containing CD4+ and CD8+ T cells and other immune cells. In addition, these biopsies often showed paucity of tumor cells or, in some cases, no tumor cells were detected in the biopsies. In the one patient assessed to date for circulating immune cells, a normalization of the CD4:CD8 ratio was seen. Analysis of longer-term imaging data to assess tumor response is in progress.

## Conclusions

Intratumoral injection of activated, autologous dendritic cells is safe and feasible. The accumulation of immune cells in tumor biopsies suggests that these injections have the potential to successfully change the tumor microenvironment from immunosuppressive to one that is conducive to the induction of anti-tumor immune responses. Normalization of the CD4:CD8 T cell ratio in circulation demonstrates the potential for systemic effects of this novel therapeutic approach.

http://ClinicalTrials.gov Identifier: NCT01882946

